# Rapid Increase in Seroprevalence of *Borrelia burgdorferi* Antibodies among Dogs, Northwestern North Carolina, USA, 2017–2021^1^

**DOI:** 10.3201/eid3010.240526

**Published:** 2024-10

**Authors:** Peyton K. Pretsch, Katherine Tyrlik-Olk, Hilary Sandborn, Dana A. Giandomenico, Alexis M. Barbarin, Carl Williams, Paul L. Delamater, Barbara Qurollo, Stephanie van der Westhuizen, Ross M. Boyce

**Affiliations:** University of North Carolina, Chapel Hill, North Carolina, USA (P.K. Pretsch, K. Tylrik-Olk, H. Sandborn, D.A. Giandomenico, P.L. Delamater, R.M. Boyce);; North Carolina Department of Health and Human Services, Raleigh, North Carolina, USA (K. Tylrik-Olk, A.M. Barbarin, C. Williams);; North Carolina State University, Raleigh (B. Qurollo);; Animal Hospital of Boone, Boone, North Carolina, USA (S. van der Westhuizen)

**Keywords:** Lyme disease, *Borrelia burgdorferi*, ticks, tick-borne disease, One Health, bacteria, vector-borne infections, zoonoses, North Carolina, United States

## Abstract

We evaluated spatial-temporal risk for Lyme disease in northwestern North Carolina, USA, by using individual-level canine *Borrelia burgdorferi* seroprevalence data collected during 2017–2021 at routine veterinary screenings for tickborne diseases. Seroprevalence in dogs increased from 2.2% (47/2,130) in 2017 to 11.2% (339/3,033) in 2021. The percentage of incident seropositivity increased from 2.1% (45/2,130) in 2017 to 7.6% (231/3,033) in 2021. Exploratory geographic analyses found canine seroprevalence shifted from clustered (2017, Moran’s *I* = 0.30) to dispersed (2021, Moran’s *I* = −0.20). Elevation, slope, aspect, and forest land cover density were associated with canine seroprevalence within various household buffer regions in 2017. Slope was associated with seroprevalence at the household level in 2021. Results support the use of individual-level canine seroprevalence data for monitoring human risk for Lyme disease. Establishing sentinel veterinary clinics within Lyme disease–emergent communities might promote prevention and control efforts and provide opportunities for educational and behavioral interventions.

Lyme disease, caused primarily by infection with *Borrelia burgdorferi*, is the most frequently reported vectorborne disease in the United States, accounting for >75% of reported tickborne diseases (*1*). *B. burgdorferi* is transmitted to both humans and animals through the bite of infected *Ixodes* spp. ticks; *I. scapularis* is the primary tick vector in the eastern United States (*2*,*3*). Early during the disease course, humans typically experience rash (i.e., erythema migrans), fever, and malaise. Untreated, the disease can progress to more severe manifestations, including carditis and arthritis (*4*). Although most infections resolve after antimicrobial drug treatment, even with early and appropriate treatment, ≈15% of persons will experience posttreatment Lyme disease syndrome, characterized by chronic pain, fatigue, and cognitive impairment often described as brain fog (*5*–*7*).

The Centers for Disease Control and Prevention records ≈30,000 Lyme disease cases per year in the United States (*8*). Those data are collected through routine surveillance systems and might greatly underestimate true infection incidence (*9*–*12*). Underreporting can be caused by empirically treated but untested or frequently unreported cases, relatively complex case definitions that vary according to transmission setting, and variations in state resources dedicated to surveillance and case investigations (*10*,*11*). In the United States, Lyme disease has historically been associated with New England and upper Midwest and mid-Atlantic regions (*3*). However, studies have suggested that northern populations of *I. scapularis* ticks are expanding their geographic range southward along the Appalachian Mountains, affecting areas such as southwestern Virginia and northwestern North Carolina (*13*–*16*). Medical providers might be less familiar with the diagnosis and management of Lyme disease in those areas, which might also contribute to underdiagnosis (*17*). Novel and more effective methods of surveillance that enable timely risk monitoring might help to overcome the prevention and awareness challenges caused by Lyme disease spread into new areas and populations. Seroprevalence among companion animals, specifically domestic dogs, has been shown to be a proxy measure for human disease risk (*18*–*21*). Although direct transmission of *B. burgdorferi* spirochetes between dogs and humans has not been reported, a pet dog would likely encounter many of the same environmental conditions that would expose humans to tick vectors (*3*,*22*). Multiple studies have shown a spatial overlap between *B. burgdorferi* seroprevalence in dogs and the incidence of human Lyme disease cases (*18*–*21*); a seroprevalence of >5% in dogs was reported to be a sensitive indicator for human infection risk (*20*).

In veterinary clinics, domestic dogs are tested for *B. burgdorferi* antibodies as part of their annual heartworm and tickborne disease screening, most commonly performed by using the SNAP 4DX Plus assay (IDEXX Laboratories, Inc., https://www.idexx.com) (*23*–*25*). SNAP assay data can provide a comprehensive and accessible mechanism for monitoring spatial-temporal changes in Lyme disease risk (*19*). However, research evaluating the trends of *B. burgdorferi* seroprevalence in individual dogs is limited because most studies have used aggregated, cross-sectional data to evaluate yearly state and county level trends (*19*,*20*). In contrast, a more detailed approach might provide crucial information that promotes timely identification of emerging risk areas, which could guide targeted educational campaigns and vector-control interventions.

The objective of this study was to evaluate the spatiotemporal risk for Lyme disease in northwestern North Carolina by using individual-level canine *B. burgdorferi* seroprevalence data. Specifically, we focused on Watauga County (Appendix Figure 1), which reported no Lyme disease cases in the 2020 North Carolina surveillance summary (*15*), despite being located in an area of emerging risk, according to entomologic and human surveillance reports (*13*,*15*,*16*,*26*). The contradictory nature of the data suggests that human cases might not have been reported or not identified and treated, potentially resulting in long-term adverse health consequences. 

## Methods

We conducted a retrospective cohort study of *B. burgdorferi* seroprevalence data collected from dogs primarily through routine screening for heartworm and tickborne disease exposure. We partnered with a large veterinary clinic that had ≈7,000 canine patients in 2021. The clinic is located in Boone, North Carolina, USA, the largest town (population ≈19,000 residents) in Watauga County (*27*). We collected data from tests completed during January 1, 2017–December 31, 2021. The University of North Carolina at Chapel Hill Institutional Review Board provided ethics approval for this study (approval no. 22–0152).

The veterinary clinic measured antibodies against *B. burgdorferi* by using the SNAP 4DX Plus assay. This point-of-care veterinary diagnostic test is an ELISA that uses the C6-peptide for detection of antibodies specifically produced during *B. burgdorferi* infections; the peptide does not cross-react with antibodies produced by canine Lyme disease vaccines (*28*,*29*). In addition, the SNAP 4DX Plus test enables simultaneous detection of *Dirofilaria immitis* antigen and antibodies against *Anaplasma phagocytophilum*, *A. platys*, *Ehrlichia canis*, and *E. ewingii* (*19*,*28*). We obtained SNAP 4DX Plus results from clinic records along with concurrent doxycycline prescriptions and client household addresses used for epidemiologic and geographic analyses.

The primary outcome measures for epidemiologic analysis were annual seroprevalence of canine *B. burgdorferi* and incident seropositivity. We defined canine *B. burgdorferi* seroprevalence as the proportion of test results positive for *B. burgdorferi* antibodies among all SNAP 4DX Plus tests completed for each year of the study period. We used incident seropositivity to measure the proportion of new *B. burgdorferi*–seropositive results that occurred among all annually completed tests and defined it as the dog’s first positive result for *B. burgdorferi* antibodies detected by the SNAP 4DX Plus test over the course of the study. Unlike measures of seroprevalence, a dog could only be incident seropositive once. We evaluated annual seroprevalence and incident seropositivity for *Anaplasma* spp. as secondary epidemiologic outcomes. We considered a dog to be *Anaplasma* spp. positive if they had a positive result for *A. phagocytophilum* or *A. platys* antibodies; the SNAP 4DX Plus test does not differentiate between the 2 species. Additional secondary outcomes were *B. burgdorferi* and *Anaplasma* spp. co-positive seroprevalence and the number of doxycycline prescriptions, which we used as an estimate for symptomatic cases among dogs with a positive test result.

To explore the spatiotemporal risk for Lyme disease, we assessed geographic clustering of canine *B. burgdorferi* seroprevalence at the household level for each year. We geocoded client household addresses through Google’s geocoding application programming interface. Then, environmental covariates were used in regression analyses to identify risk factors for annual canine *B. burgdorferi* seroprevalence during the first (2017) and last (2021) years of the study. Environmental risk factors of interest were elevation, slope, aspect, normalized difference vegetation index (NDVI), distance to green space, and forest, urban, and agricultural land cover densities. We derived elevation, slope, and aspect from the Shuttle Radar Topography Mission 30m digital elevation model using ArcGIS Pro version 3.1.2 (*30*). We derived NDVI from US Geological Survey Landsat 8 30m imagery from May 24, 2023 (*31*). NDVI is the difference between near-infrared (which vegetation reflects) and red light (which vegetation absorbs) and ranges from −1 (bare ground or water) to +1 (green vegetation). We collected land cover data from the National Land Cover Database through the Multi-Resolution Land Characteristics Consortium (*32*). For this study, forest land cover comprised deciduous, evergreen, and mixed forest classes; urban land cover comprised developed open space, low intensity, medium intensity, and high intensity classes; and agricultural land cover consisted of hay/pasture and cultivated crops classes.

We used prevalence differences (PDs) to quantify the change in annual canine *B. burgdorferi* seroprevalence and risk differences (RDs) to quantify the change in incident seropositivity for each year of the study, using 2017 as our referent year. To account for correlations that arise from repeated measures, we estimated absolute measures of effect (e.g., PD and RD) and corresponding 95% CIs by using binomial generalized estimating equations along with an identity link and robust variance estimators, assuming an exchangeable correlation structure (*33*–*37*). We iteratively estimated working correlation matrices by using all available pairs of nonmissing values in the moment estimators (*38*). We also used identical generalized estimating equations procedures and assumptions to calculate PDs to compare annual canine *Anaplasma* spp. seroprevalence and RDs to compare *Anaplasma* spp. incident seropositivity for each year, using 2017 as the referent group. We cross-sectionally evaluated the prevalence of *B. burgdorferi* and *Anaplasma* spp. co-positivity among all completed tests and the proportion of doxycycline prescriptions among positive test results for each year of the study. We used SAS version 9.4 (SAS Institute Inc., https://www.sas.com) to perform data analyses. We considered a p value <0.05 to be statistically significant.

We used Moran’s *I* statistic, aggregated to the US census block group, to evaluate whether households with similar seroprevalence of canine *B. burgdorferi* were geographically clustered in Watauga County during 2017–2021. The metric returns a value ranging from −1 to 1; the −1 indicates perfect geographic dispersion, 0 indicates perfect geographic randomness, and 1 indicates perfect geographic clustering. Furthermore, we used bivariate ordinary least squares regression to analyze the associations between environmental characteristics and *B. burgdorferi* seroprevalence for years 2017 and 2021. To account for human and dog movement, we averaged environmental variables (except distance to the nearest national, state, or local park or forest edge) across buffer regions that had radii of 0, 0.5, 1, and 3 miles around each household. We calculated the percentages of each buffer region that had forest, urban, or agricultural cover and used them as explanatory variables. In addition, we calculated the Euclidean distances in meters from each household point location to the nearest national, state, or local park or forest edge; we collected those data by using Esri (*39*). We standardized regression coefficients, enabling comparisons of coefficient magnitudes across models by using the household buffer region of 0 miles as the referent.

## Results

We identified 6,683 unique canine patients associated with 4,070 owners that had >1 completed SNAP 4DX Plus test during January 1, 2017–December 31, 2021. A total of 12,990 SNAP 4DX Plus test results were reported in clinic records. We excluded 649 SNAP 4DX Plus tests because either results were missing (n = 52) or testing was performed at a location outside of our partner clinic and those results were not accessible to study staff (n = 597), leaving 12,341 test results for inclusion in our analyses.

Over the entire study period, 914 (7.4%) tests were positive for *B. burgdorferi* antibodies, of which 690 (75.5%, 690/914) were defined as incident seropositive. Seroprevalence increased from 2.2% (47/2,130) in 2017 to 11.2% (339/3,033) in 2021 (PD = 9.39% [95% CI 8.12%–10.66%]) (Table 1). Incident *B. burgdorferi* seropositivity also increased; newly positive results increased from 2.1% (45/2,130) in 2017 to 7.6% (231/3,033) in 2021 (RD = 5.49% [95% CI 4.36%–6.62%]). However, when compared with 2017, the greatest increase in newly positive test results was observed in 2020 (RD = 5.82% [95% CI 4.61%–7.03%]).

**Table 1:** *Borrelia burgdorferi* seroprevalence and incident seropositivity among dogs screened for heartworm and tickborne diseases in Watauga County, North Carolina, USA, 2017–2021*

Year	No. tests	Seroprevalence, no. (%)	Seroprevalence difference, % (95% CI)†	Incident seropositivity, no. (%)	Risk difference, % (95% CI)†
2017	2,130	47 (2.2)	Referent	45 (2.1)	Referent
2018	2,114	77 (3.6)	1.54 (0.65–2.44)	60 (2.8)	0.71 (−0.23 to 1.65)
2019	2,447	163 (6.7)	4.83 (3.73–5.93)	145 (5.9)	3.74 (2.62–4.87)
2020	2,617	288 (11.0)	9.04 (7.76–10.31)	209 (8.0)	5.82 (4.61–7.03)
2021	3,033	339 (11.2)	9.39 (8.12–10.66)	231 (7.6)	5.49 (4.36–6.62)

*Dogs were screened by using the IDEXX SNAP 4DX Plus assay (IDEXX Laboratories, Inc., https://www.idexx.com). Seroprevalence was defined as the proportion of results positive for *Borrelia burgdorferi* antibodies among all IDEXX SNAP 4DX Plus tests completed for each year of the study. Incident seropositivity was defined as the dog’s first *Borrelia burgdorferi*–positive result during the study period. †Absolute measures of effect were analyzed by using generalized estimating equations with robust variance estimators to account to correlations that arise from repeated measures. Models assume an exchangeable correlation structure.

Among the 12,341 tests, 37 (0.3%) had a positive result for *Anaplasma* spp. antibodies, of which 35 (94.6%, 35/37) were newly positive. All *Anaplasma* spp.–positive SNAP 4DX Plus test results in 2017, 2019, and 2020 were newly positive, yielding similar seroprevalence and incident seropositivity results within each year (Table 2). Canine *Anaplasma* spp. seroprevalence increased from 0.1% (2/2,130) in 2017 to 0.4% (12/3,033) in 2021 (PD = 0.30% [95% CI 0.04%–0.55%]). The percentage of newly positive test results increased from 0.1% (2/2,130) in 2017 to 0.3% (10/3,033) in 2021 (RD = 0.24% [95% CI −0.01%–0.48%]). The largest increase occurred in 2019; newly positive results were 0.5% (RD = 0.40% [95% CI 0.09%–0.70%]). Approximately 50% of the *Anaplasma* spp.–positive results were concurrently positive for *B. burgdorferi* (51.4% [19/37]). Of the 932 dogs that had a positive SNAP 4DX Plus result for *B. burgdorferi*, *Anaplasma* spp., or both, 391 (42.0%) were provided a concurrent doxycycline prescription.

**Table 2:** *Anaplasma* spp. seroprevalence and incident seropositivity among dogs screened for heartworm and tickborne diseases in Watauga County, North Carolina, USA, 2017–2021*

Year	No. tests	Seroprevalence, no. (%)	Seroprevalence difference, % (95% CI)†	Incident seropositivity, no. (%)	Risk difference, % (95% CI)†
2017	2,130	2 (0.1)	Referent	2 (0.1)	Referent
2018	2,114	0	NA	0	NA
2019	2,447	12 (0.5)	0.40 (0.09–0.71)	12 (0.5)	0.40 (0.09–0.70)
2020	2,617	11 (0.4)	0.32 (0.05–0.60)	11 (0.4)	0.33 (0.05–0.61)
2021	3,033	12 (0.4)	0.30 (0.04–0.55)	10 (0.3)	0.24 (−0.01 to 0.48)

*Dogs were screened by using the IDEXX SNAP 4DX Plus assay (IDEXX Laboratories, Inc., https://www.idexx.com). Seroprevalence was defined as the proportion of results positive for *Anaplasma* spp. antibodies among all IDEXX SNAP 4DX Plus tests completed for each year of the study. Incident seropositivity was defined as the dog’s first *Anaplasma* spp.–positive result during the study period. A dog was considered *Anaplasma* spp. positive if it had a positive result for *Anaplasma phagocytophilum* or *Anaplasma platys* antibodies; the IDEXX SNAP 4DX Plus test does not differentiate between the 2 species. NA, not applicable. †Absolute measures of effect were analyzed by using generalized estimating equations with robust variance estimators to account for correlations that arise from repeated measures. Models assume an exchangeable correlation structure.

Exploratory geographic analysis included 2,739 client households within Watauga County. Canine *B. burgdorferi* seroprevalence in 2017 appeared higher in more population-dense areas, such as Boone and the primarily residential areas south of Boone (Figure; Appendix Figure 2). In contrast, in 2021, when canine *B. burgdorferi* seroprevalence was significantly higher, we did not observe an apparent geographic correlation. The Moran’s *I* values for canine *B. burgdorferi* seroprevalence were positive and significant for 2017 (p = 0.002), 2018 (p<0.001), and 2019 (p<0.001), indicating geographic clustering (Table 3). In the 2017 bivariate regression analysis (Table 4), elevation was positively associated with canine *B. burgdorferi* seroprevalence, and slope was negatively associated with seroprevalence in the 0.5-mile, 1-mile, and 3-mile buffer regions surrounding households. Aspect was positively associated with canine *B. burgdorferi* seroprevalence, and density of forest land cover was negatively associated with seroprevalence at the 3-mile level. In the 2021 bivariate regression analysis, only the slope was positively associated with canine *B. burgdorferi* seroprevalence at the household level.

**Figure:**
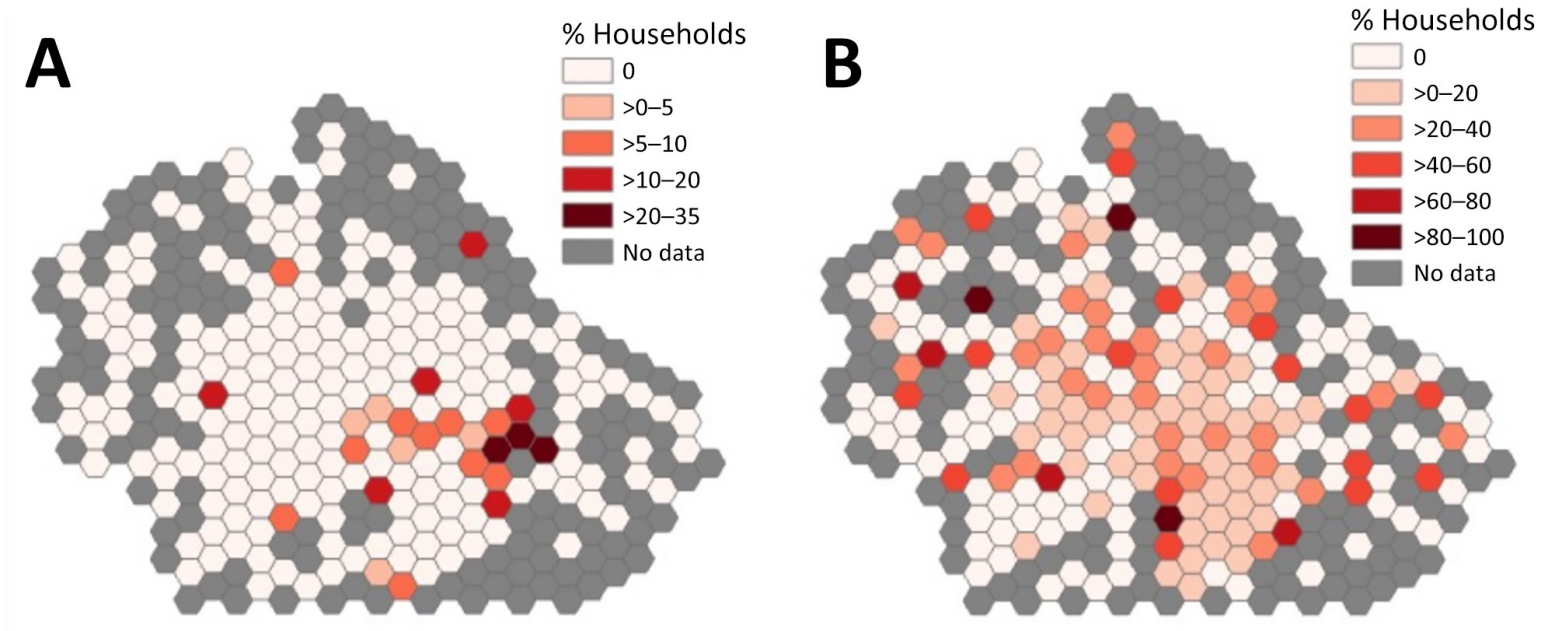
Canine *Borrelia burgdorferi* seroprevalence among surveyed households in Watauga County, North Carolina, USA, 2017–2021. Colored hexagons indicate the percentage of households with dogs positive for *B. burgdorferi* antibodies during 2017 (A) and 2021 (B). A total of 2,739 client households were included in the analysis. Seroprevalence was defined as the percentage of IDEXX SNAP 4DX Plus assay (IDEXX Laboratories, Inc., https://www.idexx.com) results that were positive for *B. burgdorferi* antibodies in dogs living within surveyed households. Lighter colors indicate areas with no or low canine *B. burgdorferi* seroprevalence; darker colors indicate areas with higher canine *B. burgdorferi* seroprevalence. Gray hexagons indicate areas with no household data.

**Table 3:** Spatial autocorrelation of canine *Borrelia burgdorferi* seroprevalence among surveyed households in Watauga County, North Carolina, USA, 2017–2021*

Year	Moran’s *I*	p value
2017	0.30	0.002
2018	0.38	<0.001
2019	0.38	<0.001
2020	0.10	0.207
2021	−0.20	0.128

*Number of surveyed households was 2,739. Moran’s *I* values were aggregated to the US census block groups (n = 35).

**Table 4:** Associations between environmental characteristics and canine *Borrelia burgdorferi* seroprevalence across various household buffer regions in Watauga County, North Carolina, USA, 2017–2021*

Characteristics	Buffer regions, 2017					Buffer regions, 2021			
	Household†	0.5-mile radius	1-mile radius	3-mile radius		Household†	0.5-mile radius	1-mile radius	3-mile radius
Elevation	−0.03 (0.03)	0.07 (0.03)‡	0.06 (0.03)‡	0.06 (0.03)‡		0.00 (0.03)	0.01 (0.03)	0.02 (0.03)	0.00 (0.03)
Slope	0.00 (0.00)	−0.06 (0.03)‡	−0.06 (0.03)‡	−0.08 (0.03)§		0.01 (0.00)‡	−0.00 (0.03)	0.02 (0.03)	−0.02 (0.03)
Aspect	−0.01 (0.06)	−0.03 (0.03)	0.04 (0.03)	0.10 (0.03)¶		−0.02 (0.05)	−0.03 (0.03)	−0.01 (0.03)	−0.01 (0.03)
NDVI	0.04 (0.03)	−0.02 (0.03)	−0.03 (0.03)	−0.04 (0.03)		0.03 (0.03)	0.01 (0.03)	0.01 (0.03)	−0.00 (0.03)
Distance to green space	−0.04 (0.03)	−0.04 (0.03)	−0.04 (0.03)	−0.04 (0.03)		0.01 (0.03)	0.01 (0.03)	0.01 (0.03)	0.01 (0.03)
Forest land cover density	−0.03 (0.03)	−0.03 (0.03)	−0.07 (0.03)‡	−0.08 (0.03)§		0.01 (0.03)	0.03 (0.03)	0.01 (0.03)	−0.05 (0.03)
Urban land cover density	0.01 (0.03)	0.01 (0.03)	0.05 (0.03)	0.05 (0.03)		−0.02 (0.03)	−0.04 (0.03)	−0.02 (0.03)	0.02 (0.03)
Agriculture land cover density	0.04 (0.03)	0.03 (0.03)	0.01 (0.03)	0.05 (0.03)		0.01 (0.03)	0.05 (0.03)‡	0.04 (0.03)	0.05 (0.03)

*Values are regression coefficients (+SE). Bivariate ordinary least squares regression was performed to determine associations; coefficients were standardized, enabling magnitude comparisons of coefficients across models by using the household level as the referent. NDVI, normalized difference vegetation index. †Household represents a buffer region radius of 0 miles. ‡p<0.05. §p<0.01. ¶p<0.001.

## Discussion

Seroprevalence and incident seropositivity of *B. burgdorferi* antibodies among domestic dogs in Watauga County, North Carolina, increased substantially from 2017 to 2021; the largest relative difference in proportions of newly positive test results occurred in 2020. Although less frequent, *Anaplasma* spp. seroprevalence and incident seropositivity also increased during 2017–2021, possibly indicating emergence of *Anaplasma spp*. in southern states. However, that result might indicate exposure to *A. platys*, which is not spread by *Ixodes* sp. ticks, unlike *A. phagocytophilum*. 

Doxycycline was prescribed for ≈50% of dogs that had a positive test result. We used concurrent doxycycline prescriptions to measure symptomatic illness; however, this might have led to an overestimate because doxycycline could have also been prescribed for asymptomatic cases. Over the study period, canine *B. burgdorferi* seroprevalence shifted from clustering in distinct geographic areas to having no distinct clusters within the county. Moreover, the observed *B. burgdorferi* seroprevalence in 2021 (11.2%) falls within the sensitivity indicator (>5%) for human infection risk (*20*) and is comparable to rates found in traditionally high-incidence states. For example, the Companion Animal Parasite Council reported an annual canine *B. burgdorferi* seroprevalence of 8.8% in Rhode Island, 11.9% in Connecticut, and 12.4% in Maine in 2021 (*40*). Therefore, our overall findings provide compelling evidence of change in canine *B. burgdorferi* seropositivity, supporting the conclusion that Lyme disease is rapidly emerging and is likely established in northwestern North Carolina (*14*).

Geographic analysis results were generally consistent with trends observed in the epidemiologic analysis. For example, in 2017, specific environmental factors were associated with canine *B. burgdorferi* seroprevalence, including elevation, slope, aspect, and density of forest land cover. This finding is consistent with previous research in southwestern Virginia that found associations between Lyme disease incidence and higher geographic elevation (*41*). In addition, others have found slope to be negatively associated with tick densities, and higher tick densities were associated with northerly aspects (*42*), consistent with our findings. Forest land cover density was negatively associated with *B. burgdorferi* seroprevalence in our study, consistent with a report that found Lyme disease risk was inversely related to forest patch area; small patches had higher risk because of a higher density of infected vectors (e.g., white-footed mouse) that thrive in fragmented forest areas (*43*). The absence of clustering or geographic associations seen in 2021 data suggests that Lyme disease risk is becoming widespread within the county without regard to specific environmental or ecologic factors.

Surveillance reports using human data have shown a similar Lyme disease trend in Watauga County; the number of reported human cases increased from 7 in 2017 to 31 in 2021 (*44*). However, well-documented limitations of traditional surveillance systems, such as underreporting, often underestimate the true risk for human infection and can, therefore, limit public health responses (*10*–*12*). Evidence of underreporting can clearly be seen during the COVID-19 pandemic, when surveillance reports from 2020 identified no human Lyme disease cases in Watauga County, despite being listed as a high-incidence North Carolina county several consecutive years before that time (*15*). This distinction is further exemplified in our findings for the year 2021, which identified 231 newly positive tests among dogs at 1 veterinary clinic in Watauga County alone compared with 31 human cases reported through traditional surveillance systems (*44*). Although data for both humans and dogs showed similar Lyme disease trends in Watauga County, monitoring changes in canine *B. burgdorferi* seroprevalence might help to overcome limitations of traditional surveillance systems by providing a more consistent, robust, and accessible data source. Furthermore, sentinel-based surveillance at veterinary clinics could be used to monitor Lyme disease risk in emergent areas at the leading edge of *Ixodes* sp. tick and *B. burgdorferi* endemicity through regular reporting of canine *B. burgdorferi* seroprevalence to local or state health departments. Observed changes in seroprevalence estimates could subsequently trigger public health interventions, such as targeted entomologic surveillance, educational efforts for clinical providers, and public awareness campaigns. Surveillance is likely to be most effective in areas where the ecology is suitable for vectors and where variations in land cover and ecologic features occur (*45*).

Most research evaluating trends in canine *B. burgdorferi* seroprevalence have used cross-sectional analysis and ecologic data (*19*,*20*). A previous observational study using data from the IDEXX Reference Laboratories network and from veterinarians who used the IDEXX VetLab Stations and software found that canine *B. burgdorferi* seroprevalence increased in North Carolina from 1.9% in 2010 to 2.3% in 2017; Watauga County was listed as 1 of the 12 counties contributing to the observed increase (*19*). Our results support and build upon those findings. However, our analysis differs because we evaluated our data on an individual level, further demonstrating the ability of canine seroprevalence to identify Lyme disease emergence into nonendemic areas, where populations might be at increased risk (*18*–*20*). By working directly with a local veterinary clinic, we obtained client addresses to further evaluate geographic risk factors associated with canine *B. burgdorferi* seroprevalence. Individual-level canine *B. burgdorferi* seroprevalence data has additional applications for human health that should be evaluated in future research. For example, knowledge of the dog’s serostatus could be used to target educational and behavioral interventions for owners of *B. burgdorferi*–positive dogs, prioritizing prevention, control, and even vaccine efforts for high-risk persons. If a human Lyme disease vaccine becomes available, veterinary clinic visits could be used as an opportunity to inform the owner about the human vaccine and discuss their risk according to their dog’s *B. burgdorferi* serostatus. This type of intervention might improve acceptance of human Lyme disease vaccines and could help counter vaccine hesitancy with nontraditional sources, such as veterinarians, providing education and individualized risk assessments to human clients (*46*,*47*).

Our study has notable strengths, including the use of a large individual-level dataset. However, the first limitation of our study is that we only collected data from 1 veterinary clinic within the county, which might limit the generalizability of our overall findings and interpretations. In addition, our results might also be less generalizable than previous studies that used publicly available data, which enabled larger scale analysis. However, we believe that the study population was representative of domestic dogs in Watauga County because our partner clinic is a large and established veterinary hospital in the community, completing >2,000 SNAP 4DX Plus tests annually. Second, the definition used to measure incident seropositivity might have included some dogs that were not tested before receiving their first positive result and, therefore, might not represent a true incident infection. However, we believe this number is low because of the relatively small proportion of dogs that tested positive during the first year of the study. Third, associated behavioral data was absent. Understanding the extent to which certain confounders influence *B. burgdorferi* seropositivity among dogs is crucial, especially among previously unexposed populations, where less is known about behavioral risk factors (*14*). For example, we did not collect information regarding the use of tick prevention or control products among dogs, which might influence a dog’s susceptibility for infection and eventually human risk for tick exposure (*48*), although we have no reason to believe that marketing or use of those products during the study period would have changed. Future research should evaluate the extent to which covariates influence exposure and outcome relationships between humans and their pet dogs to help develop and implement community awareness and prevention campaigns in areas where Lyme disease is emerging. Furthermore, some tested dogs might have had antibodies against *B. burgdorferi* that reflect exposure from other higher transmission areas for various reasons, such as relocation of owners, recreational activities outside northwestern North Carolina (e.g., hiking, hunting), and movement of animals from breeders or shelters before residing with their current owner. Not adjusting for travel history might overestimate the human Lyme disease risk in Watauga County, especially in study years before 2020, because we believe travel might have been limited because of the COVID-19 pandemic. Future research should control for this potential bias by documenting residence and travel histories of both owners and animals.

In conclusion, our findings provide support for leveraging canine *B. burgdorferi* seroprevalence in sentinel surveillance to monitor human Lyme disease risk in Lyme disease–emergent areas. Sentinel veterinary clinics might also serve as critical partners, providing opportunities for education and individualized risk assessment delivered by trusted veterinarians. Our findings in Watauga County indicate the use of canine *B. burgdorferi* seroprevalence might help overcome limitations of traditional human surveillance systems by providing more accessible and cost-effective estimates of human Lyme disease risk. 

Appendix Additional information for rapid increase in seroprevalence of *Borrelia burgdorferi* antibodies among dogs, northwestern North Carolina, USA, 2017–2021.
