# Supplementary material for: Rapid Increase in Seroprevalence of Borrelia burgdorferi Antibodies among Dogs, Northwestern North Carolina, USA, 2017–2021
Source: Emerg Infect Dis. 2024 Oct;30(10):2047–55. doi: 10.3201/eid3010.240526 (PMC11431894; doi:10.3201/eid3010.240526)
Supplement: Appendix — Additional information for rapid increase in seroprevalence of Borrelia burgdorferi antibodies among dogs, northwestern North Carolina, USA, 2017–2021. [file 24-0526-Techapp-s1.pdf]

Article DOI: https://doi.org/10.3201/eid3010.240526

EID cannot ensure accessibility for supplementary materials supplied by authors. Readers who have difficulty accessing supplementary content should contact the authors for assistance.

Rapid Increase in Seroprevalence of *Borrelia burgdorferi* Antibodies Among Dogs, Northwestern North Carolina, USA, 2017–2021

Appendix

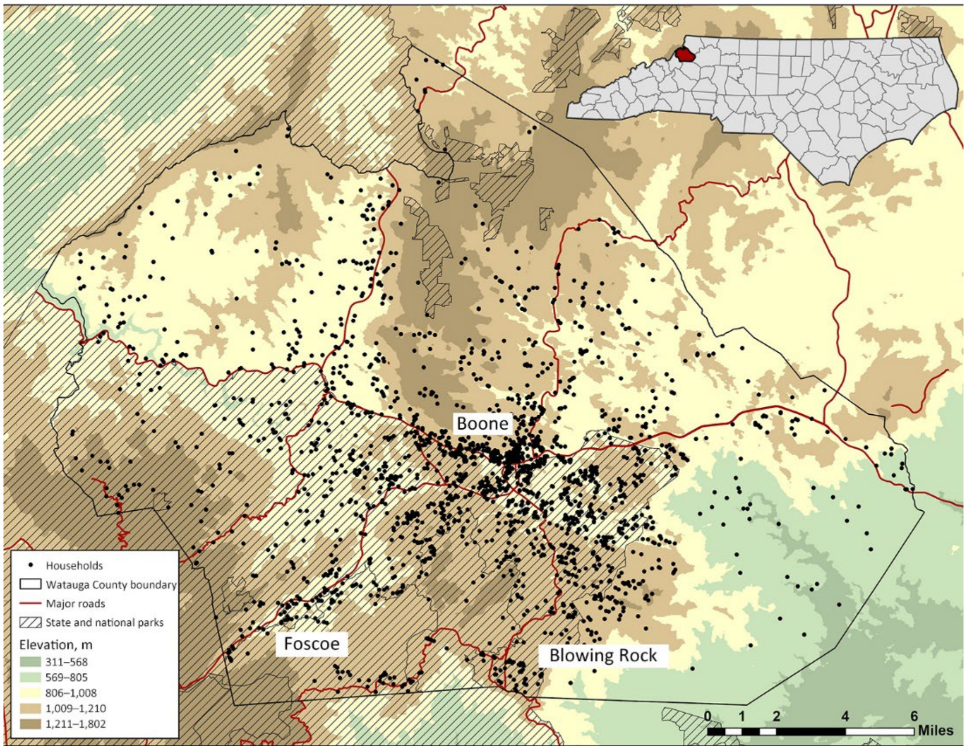

Appendix Figure 1. Elevation map of Watauga County, North Carolina, USA, displaying the location of surveyed households (n = 2,739), major roads, and state and national parks. County boundary and major road data are from the US Census Bureau (https://www.census.gov), elevation data are from the Shuttle Radar Topography Mission (https://www.earthdata.nasa.gov/sensors/srtm), and state and national park boundaries are sourced from Esri (https://www.arcgis.com/home/item.html?id=578968f975774d3fab79fe56c8c90941).

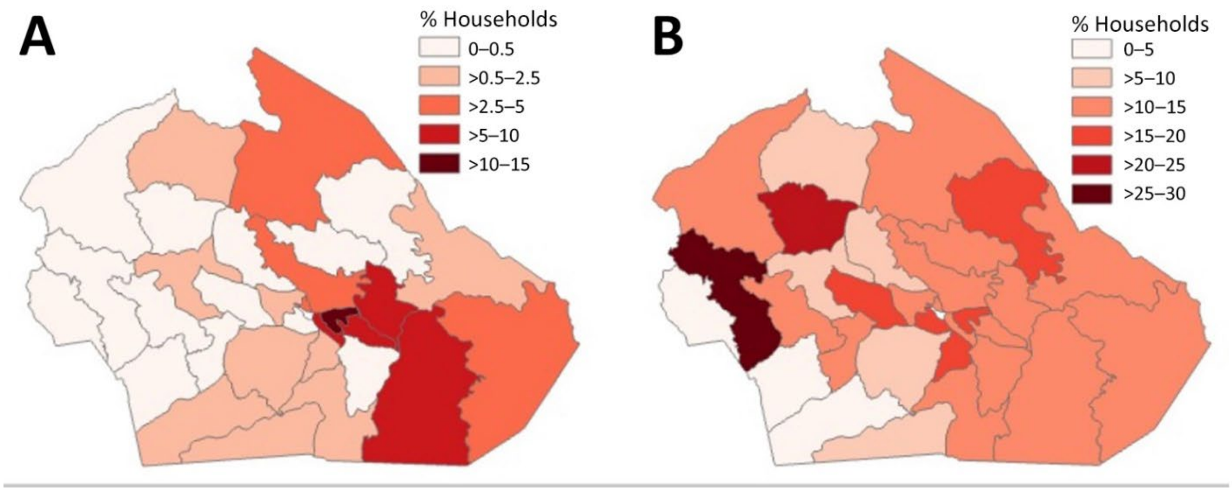

Appendix Figure 2. Canine *Borrelia burgdorferi* seroprevalence among surveyed households in Watauga County, North Carolina, 2017–2021, aggregated according to US census block groups (n = 35). Different colors indicate the percentage of households with dogs positive for *Borrelia burgdorferi* antibodies during 2017 (A) and 2021 (B) within census blocks. A total of 2,739 client households were included in this analysis. Seroprevalence was defined as the percentage of IDEXX SNAP 4DX Plus assay (IDEXX Laboratories, Inc., https://www.idexx.com) results positive for *Borrelia burgdorferi* antibodies among dogs living within surveyed households. Lighter colors indicate areas with 0 or low canine *Borrelia burgdorferi* seroprevalence; darker colors indicate areas with higher canine *Borrelia burgdorferi* seroprevalence.
